# The Effects of Combination of Coix Seed Extract and Cisplatin on TAM and Expression of HIF-1α in Vivo in Lewis Lung Carcinoma

**Published:** 2018-06

**Authors:** Guo Chen DUAN

**Affiliations:** Dept. of Fifth Oncology, The Thoracic Surgery, Hebei People’s Hospital, Shijiazhuang, Hebei 050051, P.R. China

**Keywords:** Coix seed extract, Lewis lung carcinoma, Tumor associated macrophage (TAM), Hypoxia inducible factor-1 alpha (HIF-1α)

## Abstract

**Background:**

We investigated the combined effects of Kanglaite (KLT) and cisplatin on tumor associated macrophage (TAM) and expression of hypoxia inducible factor-1 alpha (HIF-1α) in Kunming mice with Lewis lung carcinoma.

**Methods:**

Kunming mice with Lewis lung carcinoma were randomly divided into four groups: the control (NS) group, KLT group, cisplatin (DDP) group and DDP+KLT group in Hebei People’s Hospital, Hebei China from 2016 to 2017. Tumors were harvested 14 days after corresponding interventions.

**Results:**

The percentage of TAM was determined by flow cytometry and HIF-1α mRNA was detected by realtime-PCR. Tumor weight of mice in KLT group, DDP group and DDP+KLT group was significantly lower than that of NS group (*P*<0.01). Tumor growth inhibition rate in DDP+KLT group was higher than DDP group, (*P*=0.205). The spleen index was lower in the DDP group than in the NS group (*P*=0.005), but was significantly increased when combined with KLT (*P*<0.01). The percentage of TAM was higher in the DDP group than in the NS group (*P*=0.898); the combination with KLT significantly decreased the percentage (*P*=0.009<0.01). Expression of HIF-1α was lower in the KLT group and DDP+KLT group than in NS group; it was decreased more in DDP+KLT group (*P*<0.05).

**Conclusion:**

KLT led to pronounced antitumor activity in mice with Lewis lung carcinoma. It enhances the chemotherapeutic effect and improves immunity function when combined with cisplatin, which can be accomplished by decreasing the TAM levels and improving hypoxia status.

## Introduction

Lung cancer incidence ranks first in mortality due to malignant tumors in the world. Platinum based chemotherapy plays an important role in the comprehensive treatment of lung cancer, but its application is limited. Kanglaite Injection (KLT) has a synergistic effect on chemotherapy (1); however, the specific mechanism is not clear.

This article is based on the tumor associated macrophages (TAM) and hypoxia inducible factor-1α, HIF-1α, which is one of the hypoxia microenvironment markers, found in the tumor microenvironment, to study the effects and related mechanisms of KLT combined with cisplatin on mice with Lewis lung cancer.

## Materials and Methods

### Experimental materials

Healthy male Kunming mice, about 5–6 weeks old and weighing 18–20 grams, were purchased from the Hebei Experimental Animal Center (animal certificate number: 1409008). SPF class animal feeding room was provided by the clinical research center of Hebei People’s Hospital. Lewis lung cancer cell line was purchased from the Department of Immunology, Hebei Medical University and cultivated with DMEM and high glucose medium of 10% fetal calf serum, at 37 °C, in 5% carbon dioxide cell culture box. Kanglaite was purchased from Zhejiang Kanglaite Pharmaceutical Co. Ltd. (batch number: code number approved by SFDA Z10970091). Cisplatin for Injection was purchased from Qilu Pharmaceutical Co., Ltd. (specifications: 10 mg, batch No.: code number approved by SFDA H20023461). Sijiqing fetal bovine serum was from Zhejiang Tianhang Biological Technology Co. Ltd;DMEM high glucose medium was the United States Gibco Company’s product. Reverse transcriptase kit was purchased from R&D Systems, Inc. USA. PE-CD11b and FITC-F4/80 mAb were purchased from US eBioscience Company.

Ethics Committee of the hospital approved the study.

### Establishment of mouse model and intervention of grouping

After Lewis lung cancer cell strain recovery, cells were subcultured. We used Lewis lung cancer cells that exhibited good growth in 3–4 generations with trypan blue staining activity > 95%. We prepared cell suspension with cell concentration of 5×10^7^/ml; 0.2ml/piece was injected on the mice’s left anterior axillary subcutaneously. 6–7 days after inoculation, mice left anterior axillary subcutaneous palpable induration of diameter 2–3mm and the tumor model was transplanted. Vernier caliper was used to measure the longest diameter a and the shortest diameter b of the tumor on alternate days, when the longest diameter was 0.8–1cm, we abandoned the mice whose tumor was too large (ab>2.5 cm^2^) or too small (ab<0.20 cm^2^). We selected 24 tumor bearing mice with homogeneous tumor size, and they were randomly divided into NS group, KLT group, DDP group and KLT + DDP. There were 6 in each group, with intraperitoneal injection of the corresponding drugs, respectively NS/NS, KLT/NS, NS/DDP and KLT/DDP, among which, NS and KLT were intraperitoneal injected at 12.5mL/kg, DDP 1mg/Kg (the 10mgDDP soluble in 125 ml Sodium Chloride Injection) daily, twice every 12 hours, with 14 days of continuous administration.

### Tumor inhibition rate and spleen index were calculated

After the fifteenth day of administration, mice were killed and the whole tumor and spleen were removed. They were weighed respectively and the inhibition rate = (average tumor weight in the control group-average tumor volume in treatment group)/the average tumor weight in the control group×100%. Spleen index = spleen weight (mg) / body weight (g).

### TAM ratio of tumor tissue was detected by flow cytometry

The tumor tissue was prepared in the cell suspension with 2ul PE-F4/80^+^ and 2ul FITC-CD11 b^+^ antibody were added. The contents of CD11b^+^ F4/80^+^ cells were detected by flow cytometry.

### HIF-1αmRNA expression in tumor tissues was detected by Real time PCR

We extracted total cell RNA, verified integrity and purity by electrophoresis, reverse transcribed into cDNA, took real-time PCR reactions and used the RQ value for statistical analysis. GAPDH upstream primer sequence was 5′-TGAACGGGAAGCTCACTG-3′, downstream was 5′-GCTTCACCACCTTCTTGATG-3′ and the fragment length of 121bp;HIF-1αupstream primer sequence was 5′-TCAAAGTCGGACAGCCTCA-3′, downstream was 5′-CCCTGCAGTAGGTTTCTGCT-3′, with the fragment length 90bp.

### Statistical Analysis

SPSS 13.0 statistical software (Chicago, IL, USA) was used for statistical description and analysis of variance. Multiple comparison of the mean of diversity was tested by LSD-t. *P* < 0.05 indicated that the difference was statistically significant, and measurement data were expressed by with x̄±s.

## Results

### Inhibition of tumor growth in each group

**Fig. 1:**
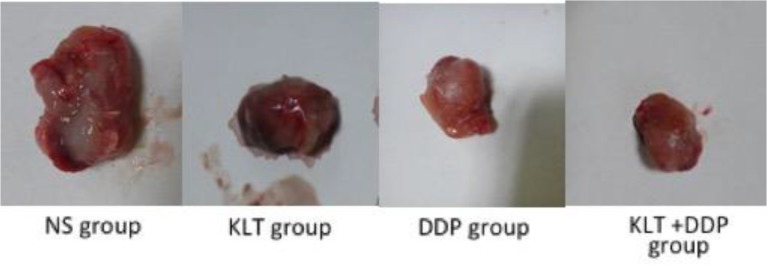
Tumor size of NS group, KLT group, DDP group and KLT +DDP group

As shown in Table I, the single and combined application of KLT and cisplatin, the tumor weight was significantly lower than in the control group; the difference was statistically significant (*P*<0.01). The tumor weight of the combined group was higher than that of the single cisplatin group, but the difference was not statistically significant (*P*=0.205). The tumor inhibition rates were 47.87%, 58.79%, 67.23%, respectively. Tumor morphology in each group was shown in Fig. 1.

**Table 1:** Tumor inhibition rate and spleen index of mice in each group

***Group***	***N***	***Tumor weight (g)***	***Tumor inhibition rate (%)***	***Spleen index (mg/g)***
NS Group	6	2.15±0.30##	-	7.31±1.50 ##
KLT Group	6	1.12±0.23**	47.87	9.70±1.53*##
DDP Group	6	0.89±0.19**	58.79	4.58±1.29*
DDP+KLT Group	6	0.71±0.23**	67.23	6.54±1.68#

# Compared with DDP group *P*<0.05

## Compared with DDP group *P*<0.01

* Compared with NS group *P*<0.05

** Compared with NS group *P*<0.01

### Effect of intervention on the spleen index of mice

As shown in Table I, the spleen index of DDP group was lower than that of the control group, KLT group, DDP+KLT group, and the difference was statistically significant (*P*<0.05). There was no significant difference in the spleen index between the control group and the DDP+KLT group (*P*=0.385). The spleen index of the two groups were higher than that of the DDP group, but lower than that of the KLT group; the differences were statistically significant (*P*<0.05).

### TAM ratio of tumor tissues in each group

As shown in Table II, compared to the control group and the KLT group, TAM ratio of tumor tissue of DDP+KLT group was decreased, and the difference was statistically significant (*P*=0.020,0.013<0.05). TAM ratio of DDP group was higher, but without statistical significance (*P*=0.898). Compared to the DDP group, the proportion of TAM in the tumor tissues of DDP+KLT group was significantly lower (*P*=0.009<0.01).

**Table 2:** The proportion of TAM in tumor tissues of each group

***Group***	***N***	***TAM (%)***
NS Group	6	6.41±1.73
KLT Group	6	4.27±1.44*#
DDP Group	6	6.52±1.77
DDP+KLT Group	6	4.08±0.67*##

# Compared with DDP group *P*<0.05

## Compared with DDP group *P*<0.01

* Compared with the control group *P*<0.05

### Expression of HIF-1αmRNA in tumor tissues of each group

**Fig. 2:**
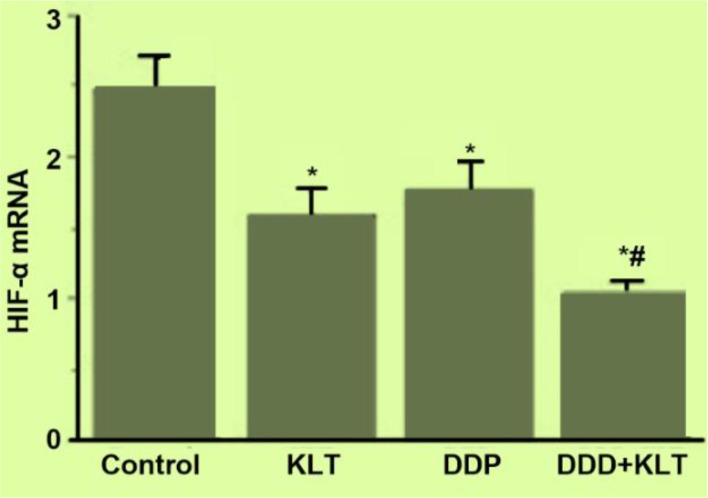
Expression of HIF-1α mRNA in tumor tissues of each group *Compared with NS group *P*<0.05 # Compared with DDP group *P*<0.05

As shown in Fig. 2, compared to the control group, the expression of HIF-1α in tumor tissue in the 3 treatment groups all decreased, and the differences were statistically significant (*P*=0.002, 0.011, 0.000<0.05). Compared to the KLT or DDP group, the DDP+KLT group was significantly lower (*P*=0.039, 0.009).

## Discussion

Lung cancer is a malignant tumor with the highest mortality rate (2). About 30–40% of lung cancer patients are diagnosed at the local advanced stage, 40% have distant metastasis, and the overall 5 year survival rate has not significantly improved. The reason is that traditional treatment mainly focuses on tumor cells themselves, and rather than the microenvironment of the tumor cells. The tumor’s microenvironment includes tumor cells and the surrounding immune cells such as fibroblasts, epithelial cells and cytokines. It is closely related to tumor development, which according to its characteristics can be divided into the hypoxic tumor microenvironment, acidic tumor microenvironment and tumor immune microenvironment.

Tumor associated macrophages (TAM) is a group of immune cells with the largest numbers in tumor immune micro environment, mainly due to differentiation and proliferation of circulating mononuclear cells. In the tumor microenvironment, TAM has the effect of immunosuppression and promoting the role of angiogenesis and tissue remodeling. Studies suggest that TAM induces a variety of growth factors such as vascular endothelial growth factor (VEGF) and transforming growth factor -β(TGF -β), to promote extracellular matrix degradation, mediate immune escape, promote blood and lymphatic vessels generation and promote tumor occurrence, development, invasion and metastasis; all this results in poor prognosis (3). TAM can also participate in chemotherapy resistance through weakening the Caspase apoptotic pathway (4), high expression of cysteine cathepsin (5), etc. The experiment shows that the KLT and cisplatin can significantly reduce the number of TAM in Lewis lung carcinoma and when combined, the number of TAM in Lewis lung carcinoma is significantly reduced, which suggests that KLT or chemotherapy alone can be used to reduce TAM in the tumor microenvironment, and can improve the tumor immune microenvironment. The combination of the two have synergistic effects. The effect of chemotherapy of KLT might be related to the decrease of TAM levels.

Hypoxic microenvironment in solid tumors has attracted more and more attention such as hypoxia-inducible factor-1 (HIF-1), which plays a central role, and the biological effect is carried out by the functional α subunit. HIF-1α, through the activation of the downstream target gene VEGF and erythropoietin, EPO, GLUT1,3, and p53 affects the angiogenesis, erythropoiesis, sugar metabolism, cell apoptosis and epithelial mesenchymal activity of tumor and surrounding tissues (6), or regulation of enzymatic activity to reduce the level of reactive oxygen species, ROS (7) to promote the development, invasion and metastasis of tumor. In this experiment, the expression of HIF-1α mRNA in the Lewis lung cancer tumor after treatment decreased, with a more significant decrease in the DDP+KLT group than in the application of KLT or DDP alone, which suggests that KLT and chemotherapeutic drugs could improve the anoxic condition by reducing HIF-1α; the combination has a synergistic effect. Lung cancer treatment methods include surgery, radiotherapy and chemotherapy, targeted therapy, immunotherapy, etc. (8). In recent years, more and more attention has been paid to traditional Chinese medicine treatment of lung cancer (9, 10). The research confirmed that Kanglaite injection had an anti-tumor effect of chemotherapeutic toxicity (1), enhancing immunity, and improving the quality of life (11). The mechanism of action may involve reducing the level of drug resistance related factors MDR1/P-gp, NF-κB/IκB, blocking mitosis, promoting tumor cell apoptosis, inhibiting angiogenesis and lymphatic vessel formation and inhibiting COX-2 expression (12). This experiment started from the perspective of tumor immune and hypoxic microenvironment. The simple application of KLT was found to inhibit tumor growth, and the spleen index significantly increased when the combined with KLT and cisplatin, suggesting that in addition to its antitumor effect, Kanglaite can also improve immune suppression induced by chemotherapeutic drugs. Similar to other reports, results of this study suggest that the anti-tumor mechanism of KLT may be achieved by improving the immune function of the body, reducing the levels of TAM in the tumor microenvironment and improving hypoxia (13, 14).

## Conclusion

KLT led to pronounced antitumor activity in mice with Lewis lung carcinoma. It enhances the chemotherapeutic effect and improves immunity function when combined with cisplatin, which can be accomplished by decreasing the TAM levels and improving hypoxia status.

## Ethical considerations

Ethical issues (Including plagiarism, informed consent, misconduct, data fabrication and/or falsification, double publication and/or submission, redundancy, etc.) have been completely observed by the authors.
